# Supplementary material for: Environment‐induced epigenetic reprogramming in genomic regulatory elements in smoking mothers and their children
Source: Mol Syst Biol. 2016 Mar 24;12(3):861. doi: 10.15252/msb.20156520 (PMC4812527; doi:10.15252/msb.20156520)
Supplement: Supplementary file 1 — Appendix [file MSB-12-861-s001.docx]

**Appendix**

Table of Contents

Materials and Methods 2

1.1 Description of the cohorts 2

1.2 Description of Discovery cohort (LINA) 2

1.3 Description of Validation cohort (LISA) 2

1.4 Isolation of gDNA from whole blood 3

1.5 Illumina WGBS library construction and sequencing 3

1.6 Sequencing library preparation by tagmentation assay 3

1.7 Whole genome bisulfite sequencing 4

1.8 Sequence alignment and cytosine methylation estimation 4

1.9 DMR calling and annotation 4

1.10 FDR estimation for DMR 5

1.11 Cellular composition estimation 5

1.12 SNP calling from WGBS data 5

1.13 Definition of genotype-DMRs 6

1.14 Pathway enrichment analysis for DMRs 6

1.15 Genomic regions enrichment 6

1.16 ChIP-seq assays 6

1.17 Peak calling for histone modification ChIP-seq 7

1.18 Genome segmentation and chromatin annotation 7

1.19 Calculation of environmentally associated chromatin state transitions between nonsmokers and smokers 8

1.20 Chromatin interaction datasets 8

1.21 lllumina RNA library preparation, sequencing and analysis 9

1.22 Pathway analysis of differentially gene expression 9

1.23 Correlation analysis between DMRs and expression of target genes 9

1.24 MassARRAY methylation analysis 9

1.25 High resolution melting for genotype assessment 10

1.26 Preparation of aqueous cigarette smoke extract (CSE) 10

1.27 Exposure of peripheral blood mononuclear cells (PBMCs) 10

1.28 RNA extraction, cDNA synthesis, and qPCR 10

1.29 Cytokine measurement 11

1.30 Murine asthma model 11

1.31 OVA-induced airway inflammation 11

1.32 Histological analysis of lung sections 12

1.33 Collection and analysis of bronchoalveolar lavage fluid-derived cells 12

1.34 Measurement of AHR 12

References 13

# Materials and Methods

## Description of the cohorts

For this study, two population-based cohorts were employed. As discovery cohort, we used the prospective mother-child cohort, LINA (**L**ifestyle and environmental factors and their **I**nfluence on **N**ewborns **A**llergy risk). For this cohort 629 mother-child pairs (622 mothers and 629 children; seven twins) were recruited between May 2006 and December 2008 in Leipzig, Germany. Blood samples were obtained from mothers at the 36^th^ week of gestation and cord blood at delivery (for details see Herberth et al, 2006; Herberth et al, 2011). Longitudinal blood samples at year one till five were taken from both children and mothers (Figure EV1). During pregnancy standardized questionnaires were recorded, as well as at each child's birthday (see also below). All questionnaires were self-administered by the parents. Maternal smoking was determined based on the questionnaire response and urine cotinine levels >100 µg/g creatinine (see also below). During annual clinical visits blood samples were obtained from children and mothers. Participation in the study was voluntary, and informed consent was obtained from all participants. The study was approved by the Ethics Committee of the University of Leipzig (046-2006, 160-2008, 160b/2008, 144-10-31052010, 113-11-18042011).

As validation cohort we used a further prospective birth cohort, LISA (Life style - Immune System - Allergy). For LISA (review board approved by the University of Leipzig, 206/2003) a total of 972 healthy neonates born in Leipzig were recruited between December 1997 and January 1999. The study design, blood sampling and the questionnaire items used for the description of children’s disease outcomes and confounding variables were comparable between LINA and LISA (see also below). Written informed consent was obtained from the parents of all children.

## Description of Discovery cohort (LINA)

During pregnancy standardized questionnaires were recorded, collecting data about smoking behavior of the parents, housing conditions, mold, traffic, noise, pets, renovation activities and personal lifestyle. Annually, starting at the child’s first birthday, disease outcomes of the children were assessed via questionnaire. Wheezing was recorded as a parental report of wheezing symptoms (“whistling or wheezing from the chest”). Children with late onset wheezing were defined according to Martinez et al. (Martinez et al, 1995) as those who had no wheezing symptoms during the first three years of life but showed wheezing symptoms after this period (at age 4 or 5). Never wheezing children did not show any wheezing or other respiratory symptoms up to the age of 5. All questionnaires were self-administered by the parents. During annual clinical visits blood samples were obtained and body weight and length evaluated.

Exposure to environmental tobacco smoke (ETS) was recorded as smoking frequency at home (‘Did you or anybody else smoke inside your dwelling during the last 12 months?’). Answering this question as ‘(almost) daily’, ‘once a week or more’ or ‘occasionally’ was defined as exposure to ETS in the subsequent analyses and ‘never’ as no exposure to ETS in the dwelling, respectively. Furthermore, the numbers of smoked cigarettes per day in the dwelling (‘How many cigarettes per day were smoked by the mother /father /anybody else in your dwelling?’) were considered.

In addition to questionnaire data, maternal urine cotinine levels were determined to assess objective smoking metabolites (Weisse et al, 2013). In concordance with these criteria mothers with a urine cotinine levels >100 µg/g creatinine and a positive answer regarding smoking during pregnancy were defined as smokers while those with a negative answer regarding smoking and a cotinine level <1 µg/g creatinine were considered as nonsmokers.

## Description of Validation cohort (LISA)

Within the prospective cohort study LISA (Life style - Immune System - Allergy) 972 healthy neonates born in Leipzig were recruited between December 1997 and January 1999. This study was designed to investigate the influence of life style and environmental factors on the maturation of the immune system and the allergy risk in early childhood. Only healthy full-term neonates of German descent were included whereas newborn children whose mothers suffered from autoimmune disease or infectious disorders during pregnancy were excluded.

Standardized questionnaires were recorded at birth and at children’s age of six, twelve and 18 months and two, four, six and ten years, collecting data about disease outcomes of the children and confounding variables such as smoking behavior of the parents, housing conditions, pets, or social status. Wheezing was recorded and defined as described for the LINA study.

For this study only samples from the sub-cohort of the city of Leipzig were included. At the age of six a clinical investigation was performed in a children hospital and whole blood samples were drawn. From 256 of the 565 children participating in the study at the age of six, DNA samples were available to investigate differential DNA methylation of the JNK2 enhancer region in the intron of GFPT2. Children’s urine samples were collected during home visits and analyzed for cotinine levels as described for the LINA study.

## Isolation of gDNA from whole blood

Maternal blood samples were collected four weeks before birth (36^th^ gestational week) and cord blood samples at birth. For longitudinal studies, six mother/child pairs were selected from the group of 16 mother/child pairs above. For those selected longitudinal subgroup, blood samples were collected at year 1 and 4 for mothers and children (Figure EV1). Genomic DNA from whole blood samples (peripheral blood or cord blood) was isolated using the QIAmp DNA Blood Mini Kit (Qiagen, Hilden, Germany), according to manufacturer’s instruction.

## Illumina WGBS library construction and sequencing

Illumina Libraries were prepared using the TruSeq DNA Sample Prep Kit v2-Set A (Illumina Inc., San Diego, CA, USA) according to the manufacturer's instructions. Briefly, 2 µg genomic DNA in 55 µl nuclease-free water (Ambion/Life Technologies GmbH, Darmstadt, Germany) was fragmented using a Covaris S2 ultrasonicator (Covaris, Woburn, Massachusetts, USA) and the following settings: 10% duty cycle, intensity 5, 200 cycles per burst, frequency sweeping, for 6 minutes. The fragmented DNA was end-repaired, extended with an 'A' base on the 3′ end and ligated with TruSeq paired-end indexing adapters. Then, adapter-ligated fragment libraries were treated with bisulfite using the EpiTect Bisulfite Kit (Qiagen, Hilden, Germany) following the instructions in the Illumina WGBS for Methylation Analysis Guide (Part # 15021861 Rev. B). After bisulfite conversion the fragment libraries were directly amplified using KAPA HiFi Uracil+ DNA Polymerase according to the settings for TruSeq™ DNA in the technical Data Sheet (KAPA HiFi HotStart Uracil+ Ready Mix, KR0413 - version 1.12, peqlab, Erlangen, Germany). Two 50 µl PCR reactions per sample were prepared and 14 cycles of PCR performed. Amplified fragment libraries were pooled and purified with 1x Agencourt AMPure XP beads (Beckman Coulter GmbH, Krefeld, Germany). WGBS Illumina Libraries were validated using Agilent 2100 Bioanalyzer (DNA 1000 Kit, Agilent Technologies) and Qubit flourometer (Qubit dsDNA HS Assay Kit, Invitrogen/ Life Technologies GmbH, Darmstadt, Germany).

The final libraries were clustered on the cBot (Illumina Inc., San Diego, CA, USA) using TruSeq PE Cluster Kit v3 according to the manufacturer`s instructions with a final concentration of either 9 pM or 10 pM (depending on the sample) spiked with 1% PhiX control v3 and an additional dedicated PhiX control lane. Sequencing on HiSeq2000 (101 bp paired-end) was performed using standard Illumina protocols and the 200-cycles TruSeq SBS Kit v3 (Illumina Inc., San Diego, CA, USA).

## Sequencing library preparation by tagmentation assay

Tagmentation-based whole genome bisulfite sequencing of sample LMCS00_004c and LMCS00_004m using about 20 ng genomic DNA as input was done as described previously with modifications. Tagmentation adapter assembly was done with oligonucleotides Tn5mC-Apt1 and Tn5mC1.1-A1block; for the oligo replacement/gap repair step, oligonucleotide Tn5mC-ReplO1 was used (Table EV10). The transposome was generated using the adapter and Tn5 transposase (Epicentre via Biozym, Hessisch Oldendorf, Germany). After oligo replacement/gap repair, the DNA was bisulfite treated using the EZ methylation kit (Zymo Research, Freiburg, Germany). Sequencing libraries were prepared with primers Tn5mCP1 and Tn5mCBar5 (LMCS00_004m) and Tn5mCBar6 (LMCS00_004c), respectively (see Table EV1A and EV10) with 12 PCR cycles on a LightCycler 480 (Roche Applied Science, Mannheim, Germany). These two libraries were sequenced on an Illumina HiSeq 2000 in the 101 bases paired-end mode.

## Whole genome bisulfite sequencing

Whole blood samples were obtained from eight smoking and eight nonsmoking mother-child pairs (see also below and Figure EV1). Illumina Libraries were prepared using the TruSeq DNA Sample Prep Kit v2-Set A (Illumina Inc., San Diego, CA, USA) according to the manufacturer's instructions. Adapter-ligated libraries were treated with bisulfite and PCR amplified (see also below). Sequencing on HiSeq2000 (101 bp paired-end) was performed using standard Illumina protocols and the 200-cycles TruSeq SBS Kit v3 (Illumina Inc., San Diego, CA, USA).

## Sequence alignment and cytosine methylation estimation

We used a mapping method as described earlier (Johnson et al, 2012) for reads from conventional whole-genome bisulfite sequencing and a modified method for reads from tagmentation-based whole-genome bisulfite sequencing. Briefly, the hg19 reference genome (37d5) was transformed *in silico* for both the top strand (C to T) and bottom strand (G to A). Before alignment, adaptor sequences were trimmed using SeqPrep (https://github.com/jstjohn/SeqPrep). Then the first read in each read pair was C-to-T converted and the 2nd read in the pair was G-to-A converted. The converted reads were aligned to a combined reference of the transformed top (C to T) and bottom (G to A) strands using BWA (bwa-0.6.1-tpx) (Li & Durbin, 2009) with default parameters except the quality threshold for read trimming (-q) of 20 and the Smith-Waterman for the unmapped mate disabled (-s). After alignment, reads were converted back to the original states and reads mapped to the antisense strand of the respective reference were removed. Duplicate reads were removed using Picard MarkDuplicates (http://picard.sourceforge.net/). Reads with alignment scores less than 1 were filtered before subsequent analysis. Total genome coverage was calculated using the total number of bases aligned from uniquely mapped reads over the total number of mappable bases in the genome.

At each cytosine position, reads that maintain the cytosine status were considered methylated, and the reads that have cytosine converted to thymine were considered unmethylated. Only bases with Phred-scaled quality score of ≥ 20 were considered. For libraries prepared with the tagmentation protocol, first 9 bp of the second read and last 9 bp before the adapter in the first read were excluded from methylation calling. Bisulfite conversion rates were estimated using the methylation level at CH sites.

## DMR calling and annotation

The bsseq v0.10 package (Hansen et al, 2012) for R statistical software v3.0.0 was used to smooth bisulfite sequencing data and call candidate DMRs. Because of our high average CpG coverage we performed smoothing on a small window size with minimum ns=11 CpGs and a minimum total width of 1 kb (h=500), breaking the smoothing if gaps between CpGs exceeded 2 kb (maxGap=2000). We calculated average raw methylation levels of each DMR and sample and performed a moderated t-test as in SAM statistics (significance analysis of microarrays (Tusher et al, 2001), R-package siggenes v1.36.0 (Schwender, 2012)) to assign p-values to each of the DMR. SAM p-values are low for DMRs that are consistently different between the groups. Based on the p-value (pSAM<0.1) and the level of mean methylation changes (∆methylation>0.1 in both raw and smoothed data), we filtered and ranked the DMR list for downstream analyses.

Initial genomic annotation of DMRs to the nearest TSS was obtained with the ‘annotatePeaks’ script of the HOMER tools software package (Heinz et al, 2010) to genome version hg19. For calculating the significance of enrichment of DMRs in certain regions of interest (ROIs), we performed a random shuffling approach. First, regions of the same size as the DMRs were randomly sampled from the whole genome. In a second step, the number of overlaps of these random regions with the ROIs was calculated using BEDTools (Quinlan & Hall, 2010). This procedure was repeated 1000 times to determine the empirical null distribution. This empirical distribution was used to estimate the one-sided upper-tail p-values for enrichment. We calculated the fold change as the number of overlaps of the DMRs with the ROIs divided by the average number as determined by the randomizations.

## FDR estimation for DMR

To estimate the false discovery rate, we considered the differentially methylated regions determined prior to the SAM filtering step (16986 regions for children and 19021 regions for mothers). Then, we performed 100 random stratifications of the samples into two groups, and determined for each of these permutations how many regions would pass the SAM filter and Δmethylation threshold. We determined the FDR to be equal to the median number of regions passing the SAM filter in the 100 randomizations, divided by the number obtained in the true smoker/non-smoker stratifications (8409 in children and 9743 in mothers). This results in estimated FDR values of 12.4% in children and 11.2% in mothers.

## Cellular composition estimation

We started out to determine representative CpG sites for 7 different blood cell subtypes (granulocytes, T-cells, CD4+ and CD8+-T-cell subsets, NK cells, B cells and monocytes) based on publically available methylation data of FACS sorted cell populations (Reinius et al, 2012). Analysis of variance was used to test beta values of each CpG in every promoter region (up to 2000bp upstream of TSS) of a particular cell type for statistical significance compared to the other cell populations considered. Post-hoc tests were applied and p-values corrected for multiple testing by Benjamini-Hochberg. Cell type specific marker regions (at least three consecutive CpGs with significant methylation differences) were ranked by distance of mean methylation in a given cell type to the mean methylation in the next closest cell type. Thereby the following characteristic sets of CpG sites were determined: granulocytes chr11:134126284, 134126323, 134126364 (*ACAD8*); CD4+ T-cells chr2:204569955, 204569976, 204570033, 204570067 (*CD28*); CD8+ T-cells chr2:87019192, 87019480, 87019492 (*CD8A*); monocytes chr22:45608465, 45608492, 45608499, 45608516 (*KIAA0930*); B cells chr19:55173482, 55173503, 55173510 (*LILRB4*) and NK cells chr12:10459912, 10459923, 10459972 (*KLRD1*).

The rationale behind our approach was that specific promoter regions of marker genes are fully demethylated in the respective cell lineage, whereas they are fully methylated for all other cell types. We calculated the average methylation level by extracting methylation levels from our WGBS at each of these CpG sites and determined (av_meth) in each sample. The proportion of the respective cell type was then estimated as 1-av_meth.

## SNP calling from WGBS data

To identify single nucleotide polymorphisms (SNPs) for each individual, we applied the bisulfite conversion aware SNP calling software Bis-SNP (Sehouli et al, 2011) using the following parameters: -U ALL; -T BisulfiteGenotyper; -C CG,1; -nt 8; -I <bam_file>; -D <dbsnp_file>; -R <reference_file>; -L <chromosome_bed_file>; -out_modes EMIT_VARIANTS_ONLY; -vfn1 <output_vcf_file>, where <bam file> is the sequence alignment file of the bisulfite converted reads, <dbsnp_file> is the vcf file from dbSNP132 (<http://www.ncbi.nlm.nih.gov/SNP/>) , containing known SNPs, <reference_file> is the 1,000 genomes phase 2 reference assembly (hs37d5) (<ftp://ftp.1000genomes.ebi.ac.uk/vol1/ftp/technical/reference/phase2_reference_assembly_sequence>) , <chromosome_bed_file> is a bed file containing the start and end positions of chromosomes 1-22, X, and Y, and <output_vcf_file> is the vcf file where the called SNPs are stored. We further filtered the resulting SNPs by a confidence score greater or equal to 50 to only obtain the most reliable polymorphisms.

## Definition of genotype-DMRs

For each DMR, all SNPs determined as previously described and located within a +/- 5kb window around the DMR were considered, following previous work on meQTL calling (Pascual et al, 2011; Pei et al, 2012). Pearson correlation between the genotype at each SNP locus within this region and the raw methylation values within the DMR were computed across all samples. To determine a null distribution, the sample labels were shuffled and the same procedure applied again. From the null distribution, we determined that the 10% FDR threshold corresponds to an absolute correlation value above 0.6. All SNPs with a higher absolute correlation value were considered as meQTLs, and all DMRs with a neighboring meQTL were considered separately and termed gDMRs (“genotype associated DMRs”)

## Pathway enrichment analysis for DMRs

Enrichment of KEGG pathways was determined for all DMRs identified in mothers (n=9743) and children (n=8409). The latest update (2013/01/30) of the online Gene SeT AnaLysis Toolkit (WebGestalt) was used to calculate statistically significant enriched pathways. Calculation of enrichment was based on a hypergeometric test followed by a Benjamini & Hochberg multiple test adjustment. A minimum of 3 genes per pathway was required to be considered for enrichment. Enrichment was considered significant at an adjusted p-value <0.05.

## Genomic regions enrichment

The enrichment analysis of DMRs within genomic regions of interest (ROIs) was done using a random sampling approach. For a given set of ROIs we randomly sampled size matched intervals from the whole genome and calculated the number of overlaps to the DMRs. This procedure was repeated 1000 times resulting in an estimate of the overlap distribution of DMRs with a random background. The mean value $\mu$ was then used to fit a Poisson distribution with $\lambda=\mu$ and probability density function $P\left(\lambda=\mu,x\right)$. A one sided test for enrichment was performed by calculating a p-value as follows: Let $n$ be the number of overlaps of DMRs with the ROI intervals, then the p-value for enrichment was calculated as

$$p_{val}=\int_{x=n}^{\infty} P\left(\lambda=\mu,x\right)$$

The odds ratio was calculated as

$$OR=\frac{n}{\mu}$$

## ChIP-seq assays

ChIP-seq of histone modifications was performed in 6 mother-child pairs including 3 smoking and 3 nonsmoking mothers and their children. Peripheral blood mononuclear cells were fixed with freshly prepared 0.4% formaldehyde in PBS for 10 min. The reaction was stopped with 125 mM glycine for 5 min. Cells were incubated in swelling buffer (25 mM HEPES, pH 7.8, 1 mM MgCl_2_, 10 mM KCl, 0.1% NP-40, 1 mM DTT) on ice for 10 min. Then the cells were resuspended in MNase buffer (25 mM KCl, 4 mM MgCl_2_, 1 mM CaCl_2_, 50 mM Tris/HCl pH 7.4) and 4 U MNase per 1x10^6^ cells was added. After 15 min incubation at 37°C, MNase was stopped by adding 10x covaris buffer (100 mM Tris pH 8.0, 2 M NaCl, 10 mM EDTA, 5% N-lauroylsarcosine, 1% Na-deoxycholate, supplemented with protease inhibitors). The samples were sonicated for 15 min with a Covaris S2 system with the following parameters: burst 200, cycle 20%, intensity 8. After centrifugation the supernatant was collected and directly used for IP.

After IgG preclearance the sheared chromatin was incubated with protein G magnetic beads (Cell signaling, 9006) and with 4 µg of the following antibodies over night: H3K4me1 (abcam, ab8895), H3K9me3 (abcam, ab8898), H3K27me3 (abcam, ab6002), H3K27ac (abcam, ab4729) and H3 (abcam, ab1791). After washes with 1x covaris buffer (10 mM Tris–HCl, pH 8.0, 200 mM NaCl, 1 mM EDTA, 0.5% N-lauroylsarcosine, 0.1% Na–deoxycholate), high-salt-buffer (50 mM Hepes pH 7.9, 500 mM NaCl, 1mM EDTA, 1% Triton X-100, 0.1% Na–deoxycholate, 0.1% SDS), lithium buffer (20 mM Tris–HCl pH 8.0, 1 mM EDTA, 250 mM LiCl, 0.5% NP-40, 0.5% Na–deoxycholate) and 10 mM Tris–HCl, chromatin was eluted from the magnetic beads (elution buffer: 50 mM Tris pH 8.0, 1 mM EDTA, 1% SDS, 50 mM NaHCO_3_) and the crosslink was reversed over night. After RNase A and proteinase K digestion, DNA was purified and cloned in a barcoded sequencing library for the Illumina sequencing platform. In brief, after DNA repair and A-addition NEBNext adapters (NEB, E7335) were ligated and digested with the USER enzyme. Barcodes (NEB, E7335) were introduced via PCR with a maximum of 14 cycles by the NEBNext polymerase (NEB, M0541). Size selection for mononucleosomal insert fragments was done with Ampure XP beads (Agencourt, A63880).

## Peak calling for histone modification ChIP-seq

Regions of the genome exhibiting significant enrichment of histone modifications were identified using SICER v1.3 and MACS v 1.4.1 . Reads were aligned as outlined by Feng et al. . Briefly, reads were aligned to the GRCh37 human genome assembly with hs37d5 decoy sequence concatenated using the bowtie 1.0 short read aligner (Liu et al, 2012) only reporting the uniquely mapping reads in SAM format (Gutierrez-Arcelus et al, 2013). MACS was used to call peaks setting the histone modification SAM file as the treatment and the H3 SAM file as the control files, suppressing calculation of shifting model, using a fixed background lambda, and using the shift size as half the size reported by the Bioanalyzer (or 166 bp, the mean fragment size, in the absence of a reported fragment size). A threshold of p <= 1e-5 was used to identify significant peaks. Additional peaks were called user SICER. The SAM files were sorted using samtools sort, and then converted to BED format using bedtools bamToBed (Zang et al, 2009). SICER was used to call peaks on all histone modifications using H3 as a control, removing all duplicate reads, defining the fragment length as in MACS, 0.85 expected genome coverage, a window size of 200bp, gapping enriched windows 200bp apart, and setting the FDR cutoff to 0.05. The MACS and SICER peaks calls were merged to maximize sensitivity. The peak-calling summary can be seen in Table EV1B. Quality control statistics were calculated, as outlined by Landt et al. (Zhang et al, 2008) including the fraction of read in peaks (FRiP), PCR bottleneck coefficient (PBC) using custom scripts, and the normalized and relative strand correlations (NSC/RSC) using SPP v1.046 in R v2.15.0 ([http://www.R-project.org](http://www.R-project.org/)).

## Genome segmentation and chromatin annotation

The chromatin was segmented and annotated using a multivariate Hidden Markov Model, ChromHMM (Feng et al, 2012). We trained a ChromHMM model over the four histone modification marks (H3K4me1, H3K27ac, H3K27me3 and H3K9me3) across all samples, which was subsequently used to segment the genome of each individual. The model was learned using the merged peak calls from MACS and SICER as the binarized input and allowing for a maximum of 400 iterations. We generated models with 5-16 states, and decided to use 16 states as this captures all combinations of the four histone modifications so that rare chromatin states are also represented (Figure 4B). Each chromatin state was labeled based on a biologically interpretable name referred to the co-occurring histone marks. Chromatin states were merged over subsets of the data covering mothers, children and their smoking and nonsmoking subsets, where at least two samples had a genomic locus labeled to be of a particular chromatin state. To annotate promoter associated marks, we identified all features overlapping with a RefSeq TSS, directly neighboring the overlapping feature with the TSS and its direct neighbors, and all features 400 bp from these, and annotated them as “TSS associated”. The remaining was labeled as “not TSS associated”. Active regulatory elements were identified by merging three active states (States 1, 2 and 3, see Figure 4) into a common meta-state. Active regulatory elements, which were not TSS associated, were defined as enhancers. A repressed meta-state was defined by merging states 12, 13 and 14.

## Calculation of environmentally associated chromatin state transitions between nonsmokers and smokers

For children and mothers, at each variable site, the state matrix of nonsmokers vs. smokers was recorded to build a chromatin state transition matrix between smokers and nonsmokers, where each observed transitions between a nonsmoker sample and smoker sample accounted for a relative proportion of all the nonsmoker samples vs. smoker samples state transitions at that site. A variable site was defined as a 200 bp window where at least in both smokers or nonsmokers there was at least a single state which occurred twice, where the recurrent state in smokers was not the same as the recurrent state in nonsmokers. The transitions “to active” were calculated as the transition of any non active state to an active state (states 1, 2 and 3) in smokers, and the transition of state 2 (active 2, H3K27ac only) and state 3 (poised, H3K4me1 only) to state 1 (active 1, H3K27ac and H3K4me1) which would represent state changes from an active to a more active state. Likewise, the transitions “to repressed” were calculated as the transition of any non repressed state to a repressed state (states 12, 13 and 14) and the transition of state 12 (PRC repressed, H3K27me3 only) and state 13 (hetero repressed, H3K9me3 only) to state 14 (highly repressed, H3K27me3 and H3K9me3) which would represent state changes from a repressed to a more repressed state. The transitions “to bivalent” were calculated as the transition of any non-bivalent state to a bivalent state (states 4 - 11). The inter-bivalent transitions were not considered, as it was difficult to assign ranks of relative “bivalentness” between the chromatin states.

The enrichment and significance of genome wide transitions was calculated from generating an empirical null normal distribution from all the non redundant permutations (n=68) of shuffling the nonsmoker and smokers as a random group of 4 samples vs. 4 sample, where each group would contain at least 1 smokers and 1 nonsmoker.

The enrichment and significance of the transitions in the DMRs, gDMRs and ngDMRs was calculated from generating an empirical null normal distribution of from 1000 random shuffles of the DMR/gDMR/ngDMR regions using shuffleBed from the bedtools suite.

## Chromatin interaction datasets

We used two different published datasets describing genomic interactions between distal loci. First, a dataset of interactions obtained from ChIA-PET, downloaded from the UCSC/ENCODE website (http://genome.ucsc.edu/ENCODE/downloads.html). We merged the datasets for various cell lines (MCF7, K562, HCT116, HeLa and NB4) in order to compile an extensive list of potential interactions across different conditions. An interaction between a genomic element and a gene was defined as any event in which a genomic locus overlaps with one of the ChIA-PET loci, while the paired locus overlaps with a region located -1kb/0kb from the TSS of UCSC known genes. The second dataset is based on predicted interactions obtained from DNAseI hypersensitivity assays conducted as part of the ENCODE project in 125 cell lines (Thurman et al., 2012). The predicted interactions relate distal DHS regions with gene promoters. An interaction between a genomic element and a gene was defined as any event in which the DHS overlaps with the genomic element considered. The dataset was downloaded from <ftp://ftp.ebi.ac.uk/pub/databases/ensembl/encode/integration_data_jan2011/byDataType/openchrom/jan2011/dhs_gene_connectivity/>

## lllumina RNA library preparation, sequencing and analysis

RNA-seq libraries were constructed using the Illumina TruSeq RNA Sample Preparation Kit v2 according to the manufacturer's instructions. Briefly, mRNA is polyA-selected from 100 – 1000 ng of total RNA and fragmented. Then first-strand cDNA was synthesized with random hexamer and SuperScript II (Invitrogen). After second-strand synthesis, the double-stranded cDNA fragments were end-repaired, A-tailed on the 3′ end, ligated to indexed adapters and amplified with 12 cycles of PCR. The final libraries were validated using Qubit (Invitrogen) and Agilent 2100 Bioanalyzer (Agilent Technologies). Subsequently, the libraries were diluted to 10nM and pooled in equimolar ratios. The pooled libraries (2 plex) were clustered on the cBot (Illumina) according to the manufacturer`s instructions with a final concentration of 10 pM spiked with 1% PhiX control v3 using TruSeq PE Cluster Kit v3. Sequencing on Illumina HiSeq2000 (101 bp paired-end) was performed using standard protocols and the 200-cycles TruSeq SBS Kit v3.

RNA sequences were aligned to hg19 reference genome using the STAR alignment software (Dobin et al, 2013), with Gencode v19 as the transcriptome annotation. Counts of reads mapped to exons were estimated by htseq-count (Anders et al, 2015). For downstream analysis, raw counts were normalized by the Voom method in the limma package (Ritchie et al, 2015) and the Combat method in the sva package (Leek & Storey, 2007) was applied to correct for possible batch effects.

## Pathway analysis of differentially gene expression

Over-represented pathways in target genes of DMRs were determined using WebGestalt. All genes belonging to one of those pathways were collected, and the t-value for the smoking vs. nonsmoking samples was computed over all genes in the pathway using the normalized expression data from RNA-seq. The t-value of the pathway was then defined as: T_P_ = sum(abs(t_i_)), where the index runs over all genes in the pathway that are targeted by a DMR.

To compute an empirical p-value for the observed statistics, the smoking/nonsmoking labels were randomly shuffled 1000 times, and the corresponding T_P_ value was computed for each randomization. The p-value was defined as the number of randomizations with a T_P_ value greater than the observed value, divided by 1000.

## Correlation analysis between DMRs and expression of target genes

We calculated the Spearman correlation between the mean DMR methylation and the target gene expression. For DMRs containing multiple CpG sites, mean methylation across CpG sites were calculated per sample. DMRs were first intersected with different chromatin states inferred from ChIP-seq data. For DMRs overlapping with void or TSS-associated states, the closest gene was used as the target gene. For DMRs overlapping with repressed or non-TSS-associated states, we used the predicted target gene from public interaction datasets (ChIA-PET and promoter-DHS interactions, see also below). For DMEs (DMRs overlapping with enhancers), we used the union of the target genes of the DMR and the overlapping enhancer. For each DMR-target gene pair, the significance of the correlation was calculated by the Spearman correlation test and the cutoff for significance was set to 0.05.

## MassARRAY methylation analysis

Quantitative DNA methylation analyses of the enhancer in the GFPT2 and TMEM241 gene were performed using Sequenom’s MassARRAY platform. Briefly, genomic DNA from whole blood samples was chemically modified with sodium bisulfite using the EZ methylation kit (Zymo Research, Freiburg, Germany) according to the manufacturer’s instructions. PCR primers were designed with an additional T7 promoter tag for *in vitro* transcription for each reverse primer, as well as a 10-mer tag on the forward primer. Bisulfite treated DNA was PCR amplified (forward primer GFPT2: aggaagagagGGTTTTTTTTATTTTGGTGTTG, reverse primer GFPT2: cagtaatacgactcactatagggagaaggctTACCACAAATAATAACACACC, region of interest: chr5:179740830-179741161; forward primer TMEM241: aggaagagagTTATGTTTGGTTTATAGTATTAG, reverse primer TMEM241: cagtaatacgactcactatagggagaaggctTATACTACCCCTACACTATAC, region of interest: chr18:21002501-21002763) using HotStarTaq DNA Polymerase (Qiagen, Hilden, Germany) with the following cycling program: 95 °C for 15 min, followed by 45 cycles of 94 °C for 30 sec, 60 °C for 30 sec, 72 °C for 1 min and a final elongation step at 72 °C for 5 min on a LightCycler 480 (Roche Applied Science, Mannheim, Germany). The PCR product was *in vitro* transcribed and cleaved by RNase A using the EpiTyper T Complete Reagent Set (Sequenom, Hamburg, Germany) and subjected to MALDI-TOF mass spectrometry analysis to determine methylation patterns as previously described. DNA methylation standards (0%, 20%, 40%, 60%, 80%, and 100% methylated genomic DNA) were used to control for potential PCR bias.

## High resolution melting for genotype assessment

High-resolution melting (HRM) analysis was performed to evaluate the genotype of rs55901738 in children (n=473) and mothers (n=618) of the LINA cohort and a subset of children (n=273) of the LISA cohort.

Based on 10ng of gDNA the genomic region chr5:179,741,265-179,741,400 was PCR amplified (forward primer: 5’-caaagccatacagctggtga-3, reverse primer: 5’-aacaccacttggggacattc-3’) using the LightCycler® 480 High Resolution Melting Master (Roche Diagnostics, Mannheim, Germany) with a final concentration of 3.5mM MgCl_2_. PCR was carried out with the following cycling program: 95°C for 10 min, 45 cycles of 95°C for 20 sec, 65°C for 30 sec (touchdown to 59°C, steps: 0.6°C), 72°C for 30 s, followed by touch-down HRM from 78°C to 95°C. The genotype was determined using LightCycler® 480 Gene Scanning software (Roche Diagnostics).

## Preparation of aqueous cigarette smoke extract (CSE)

CSE was prepared according to the protocol described by Adenuga et al. (Langmead & Salzberg, 2012). Briefly, a research-grade reference cigarettes (3R4F) from the University of Kentucky (Tobacco Health Research, Lexington, KY) were used to prepare cigarette smoke extract (CSE) by slowly bubbling smoke from one cigarette into 10 ml of RPMI 1640 without supplements at a rate of 1 cigarette/minute. Afterwards CSE was sterile-filtered through a 0.22-μm filter (Sartorius, Göttingen, Germany).

## Exposure of peripheral blood mononuclear cells (PBMCs)

Buffy coats from healthy donors were obtained from the local blood bank (Universitätsklinikum Leipzig, Germany). PBMCs were isolated by density centrifugation using Ficoll-Paque™ PLUS (GE Healthcare) and cryopreserved in liquid nitrogen. Thawed PBMCs were seeded at 4x10^5^ cells/ml in RPMI supplemented with 10 % FCS, 1 % Alanin/Glutamin, 1 % Penicillin-Streptamicin, 10 mM HEPES, and 2 µg/ml anti-CD3/anti-CD28 antibody and exposed to cigarette smoke extract (1:10^6^ dilution) or medium alone for 4 days. Genomic DNA was isolated using QIAamp DNA Blood Mini Kit (Qiagen, Hilden, Germany) following manufacturer’s instruction. Isolated gDNA was bisulfite converted and analyzed by MassARRAY technique (see MassARRAY methylation analysis).

## RNA extraction, cDNA synthesis, and qPCR

Total RNA was prepared from fresh cord blood by using peqGold RNA Pure (peqlab, Erlangen, Germany) and from in PAXgene Blood RNA Tube collected blood of year 4 by PAXgene Blood RNA Kit (Qiagen, Hilden, Germany), according to manufacturer’s instructions. The cDNA synthesis was carried out with 5 µg of RNA by using ImProm-II^TM^ Reverse Transcription System (Promega, Mannheim, Germany).

Gene expression was measured using the 96.96 Dynamic Array or FLEXsix Integrated Fluidic Circuits (IFCs) (Fluidigm, San Francisco, CA, USA). Intron-spanning primers were designed and UPL probes were selected by the Universal Probe Library Assay Design Center (http://qpcr.probefinder.com/organism.jsp, GFPT2_for 5’gctttttcatcagccagtca, -rev 5’ccgcggtccttacagtagc, UPL 79; JNK2_for 5’tgcgtcacccatacatcact, -rev 5’ ttcttccaactgggcatcat, UPL 77). A preamplification reaction was performed by pooling all primers (final concentration, 50 nM), 5 µl of cDNA and 2x PreAmp Master Mix (Applied Biosystems/Life Technologies GmbH, Darmstadt, Germany). The cycling program consisted of 95°C for 10 min, followed by 14 cycles of 95°C for 15 sec and 60°C for 4 min on a LightCycler 480 (Roche Applied Science, Mannheim, Germany). The qPCRs of 1:5 diluted with TE buffer preamplified templates were performed following manufacture’s instruction for UPL (Roche Applied Science, Mannheim, Germany) assays. Briefly, for each individual assay, a 10X Assay Mix that contained 2 µM of each forward and reverse primer, 1 µM UPL probe and 0.025% Tween-20 was prepared, and 5 µl of the mix was loaded into the assay inlets of the array. Into the sample inlets, 5 µl of the following solution was dispensed: 2.5 µl of PreAmp sample in 1.1X of FastStart Universal Probe Master Mix (Roche Applied Science, Mannheim, Germany). The cycling program consisted of 2 min at 50°C, 10 min at 95°C, followed by 35 cycles of 95°C for 15 sec, 70°C for 5 sec, and 1 min at 60°C. All reactions were performed in triplicates.

Gene expression values were determined by using the 2^-∆∆CT^ method with *GAPD* and *GUSB*, as reference genes and normalized to the lowest measured value.

## Cytokine measurement

Heparinized blood samples from mother-child pairs were obtained by venipuncture and processed within six hours for further analysis. After incubating for 4 h at 37°C, samples were diluted with RPMI-1640 medium without supplements in a ratio of 1:1 and centrifuged. Cell-free supernatants were collected and stored at -80°C until subsequent analysis. IL-8 concentration was determined in the supernatants of whole blood samples by flow cytometry using the BD CBA Human Soluble Flex Set system (BD Bioscience, Heidelberg, Germany) according to the manufacturer’s instructions and as described previously (Li & Durbin, 2009).

In brief, cytokine specific antibody coated beads were incubated for 1 h with 25 µl of blood samples or standard solution. Thereafter, samples were incubated with the corresponding PE labeled detection antibodies for 2 h. After one washing step samples were measured by flow cytometry. Analyses of data and quantification of cytokines were performed using the FCAP Array^TM^ software (Becton Dickinson, Heidelberg, Germany) on the basis of corresponding standard curves. Finally, the plasma dilution factor was accounted for.

## Murine asthma model

Mice were bred an maintained at the animal facility of the Tokyo Medical University. All experiments were approved by the Ethics Committee of Animal Experiments of the Tokyo Medical University. JNK2^-/-^ and C57BL/6J wild type (WT) mice were sensitized and challenged with ovalbumin (OVA) and assayed for airway inflammation and airway hyperreactivity (AHR) as described before (Quinlan & Hall, 2010).

## OVA-induced airway inflammation

JNK2^-/-^ and WT mice were sensitized with 10 μg OVA without alum (Sigma, St. Louis, MO, USA) in 0.2 mL PBS intraperitoneally on days 0, 3, 5, 7, 9, 11, and 13. Control mice received the same volume of PBS. All groups of mice were challenged with 200 μg OVA or PBS intranasally on days 31, 34, and 37.

## Histological analysis of lung sections

Lungs were inflated with 20% formaldehyde, and embedded in paraffin. The blocks were sectioned and stained with Haematoxylin & Eosin (HE) or with Periodic-Acid-Schiff (PAS) according to standard protocols.

## Collection and analysis of bronchoalveolar lavage fluid-derived cells

Mouse airways were lavaged three times with 1.0 mL PBS containing 2% fetal calf serum (FCS) via a tracheal cannula. The bronchoalveolar lavage (BAL) fluid was centrifuged, and cells were recovered in 0.5 mL PBS containing 2% FCS. Differential cell counts (for macrophages, lymphocytes, neutrophils, and eosinophils) were obtained using a Sysmex XT-2000iv automated cell counter (Sysmex Corporation, Kobe, Japan).

## Measurement of AHR

Airway responsiveness was assessed by inducing airflow obstruction with a methacholine aerosol using an invasive method as previously described (Takada et al., 2013). Briefly, *in vivo* lung resistance (RL) was measured in mice that were anesthetized with xylazine (10 mg/kg body weight)/ketamine (100 mg/kg body weight), tracheotomized and connected to a ventilator. Mice were ventilated with a tidal volume of 0.25 ml and 2 cm H_2_O positive end-expiratory pressure. Baseline RL and responses to aerosolized PBS were measured first, followed by responses to increasing doses (3.125 to 50 mg/ml) of aerosolized methacholine.

# References

Anders S, Pyl PT, Huber W (2015) HTSeq--a Python framework to work with high-throughput sequencing data. *Bioinformatics* **31:** 166-169

Dobin A, Davis CA, Schlesinger F, Drenkow J, Zaleski C, Jha S, Batut P, Chaisson M, Gingeras TR (2013) STAR: ultrafast universal RNA-seq aligner. *Bioinformatics* **29:** 15-21

Feng J, Liu T, Qin B, Zhang Y, Liu XS (2012) Identifying ChIP-seq enrichment using MACS. *Nat Protoc* **7:** 1728-1740

Gutierrez-Arcelus M, Lappalainen T, Montgomery SB, Buil A, Ongen H, Yurovsky A, Bryois J, Giger T, Romano L, Planchon A, Falconnet E, Bielser D, Gagnebin M, Padioleau I, Borel C, Letourneau A, Makrythanasis P, Guipponi M, Gehrig C, Antonarakis SE et al (2013) Passive and active DNA methylation and the interplay with genetic variation in gene regulation. *eLife* **2:** e00523

Hansen KD, Langmead B, Irizarry RA (2012) BSmooth: from whole genome bisulfite sequencing reads to differentially methylated regions. *Genome Biol* **13:** R83

Heinz S, Benner C, Spann N, Bertolino E, Lin YC, Laslo P, Cheng JX, Murre C, Singh H, Glass CK (2010) Simple combinations of lineage-determining transcription factors prime cis-regulatory elements required for macrophage and B cell identities. *Molecular cell* **38:** 576-589

Herberth G, Daegelmann C, Weber A, Roder S, Giese T, Kramer U, Schins RP, Behrendt H, Borte M, Lehmann I (2006) Association of neuropeptides with Th1/Th2 balance and allergic sensitization in children. *Clinical and experimental allergy : journal of the British Society for Allergy and Clinical Immunology* **36:** 1408-1416

Herberth G, Hinz D, Roder S, Schlink U, Sack U, Diez U, Borte M, Lehmann I (2011) Maternal immune status in pregnancy is related to offspring's immune responses and atopy risk. *Allergy* **66:** 1065-1074

Johnson MD, Mueller M, Game L, Aitman TJ (2012) Single nucleotide analysis of cytosine methylation by whole-genome shotgun bisulfite sequencing. *Current protocols in molecular biology / edited by Frederick M Ausubel [et al]* **Chapter 21:** Unit21.23

Langmead B, Salzberg SL (2012) Fast gapped-read alignment with Bowtie 2. *Nat Methods* **9:** 357-359

Leek JT, Storey JD (2007) Capturing heterogeneity in gene expression studies by surrogate variable analysis. *PLoS genetics* **3:** 1724-1735

Li H, Durbin R (2009) Fast and accurate short read alignment with Burrows-Wheeler transform. *Bioinformatics* **25:** 1754-1760

Liu Y, Siegmund KD, Laird PW, Berman BP (2012) Bis-SNP: combined DNA methylation and SNP calling for Bisulfite-seq data. *Genome Biol* **13:** R61

Martinez FD, Wright AL, Taussig LM, Holberg CJ, Halonen M, Morgan WJ (1995) Asthma and wheezing in the first six years of life. The Group Health Medical Associates. *The New England journal of medicine* **332:** 133-138

Pascual M, Suzuki M, Isidoro-Garcia M, Padron J, Turner T, Lorente F, Davila I, Greally JM (2011) Epigenetic changes in B lymphocytes associated with house dust mite allergic asthma. *Epigenetics : official journal of the DNA Methylation Society* **6:** 1131-1137

Pei L, Choi JH, Liu J, Lee EJ, McCarthy B, Wilson JM, Speir E, Awan F, Tae H, Arthur G, Schnabel JL, Taylor KH, Wang X, Xu D, Ding HF, Munn DH, Caldwell C, Shi H (2012) Genome-wide DNA methylation analysis reveals novel epigenetic changes in chronic lymphocytic leukemia. *Epigenetics : official journal of the DNA Methylation Society* **7:** 567-578

Quinlan AR, Hall IM (2010) BEDTools: a flexible suite of utilities for comparing genomic features. *Bioinformatics* **26:** 841-842

Reinius LE, Acevedo N, Joerink M, Pershagen G, Dahlen SE, Greco D, Soderhall C, Scheynius A, Kere J (2012) Differential DNA methylation in purified human blood cells: implications for cell lineage and studies on disease susceptibility. *PloS one* **7:** e41361

Ritchie ME, Phipson B, Wu D, Hu Y, Law CW, Shi W, Smyth GK (2015) limma powers differential expression analyses for RNA-sequencing and microarray studies. *Nucleic acids research* **43:** e47

Schwender H (2012) siggenes: Multiple testing using SAM and Efron's empirical Bayes approaches, R package version 1.36.0.

Sehouli J, Loddenkemper C, Cornu T, Schwachula T, Hoffmuller U, Grutzkau A, Lohneis P, Dickhaus T, Grone J, Kruschewski M, Mustea A, Turbachova I, Baron U, Olek S (2011) Epigenetic quantification of tumor-infiltrating T-lymphocytes. *Epigenetics : official journal of the DNA Methylation Society* **6:** 236-246

Tusher VG, Tibshirani R, Chu G (2001) Significance analysis of microarrays applied to the ionizing radiation response. *Proceedings of the National Academy of Sciences of the United States of America* **98:** 5116-5121

Weisse K, Winkler S, Hirche F, Herberth G, Hinz D, Bauer M, Roder S, Rolle-Kampczyk U, von Bergen M, Olek S, Sack U, Richter T, Diez U, Borte M, Stangl GI, Lehmann I (2013) Maternal and newborn vitamin D status and its impact on food allergy development in the German LINA cohort study. *Allergy* **68:** 220-228

Zang C, Schones DE, Zeng C, Cui K, Zhao K, Peng W (2009) A clustering approach for identification of enriched domains from histone modification ChIP-Seq data. *Bioinformatics* **25:** 1952-1958

Zhang Y, Liu T, Meyer CA, Eeckhoute J, Johnson DS, Bernstein BE, Nusbaum C, Myers RM, Brown M, Li W, Liu XS (2008) Model-based analysis of ChIP-Seq (MACS). *Genome Biol* **9:** R137
