# Supplementary material for: Environment‐induced epigenetic reprogramming in genomic regulatory elements in smoking mothers and their children
Source: Mol Syst Biol. 2016 Mar 24;12(3):861. doi: 10.15252/msb.20156520 (PMC4812527; doi:10.15252/msb.20156520)
Supplement: Supplementary file 2 — Expanded View Figures PDF [file MSB-12-861-s002.pdf]

Expanded View Figures

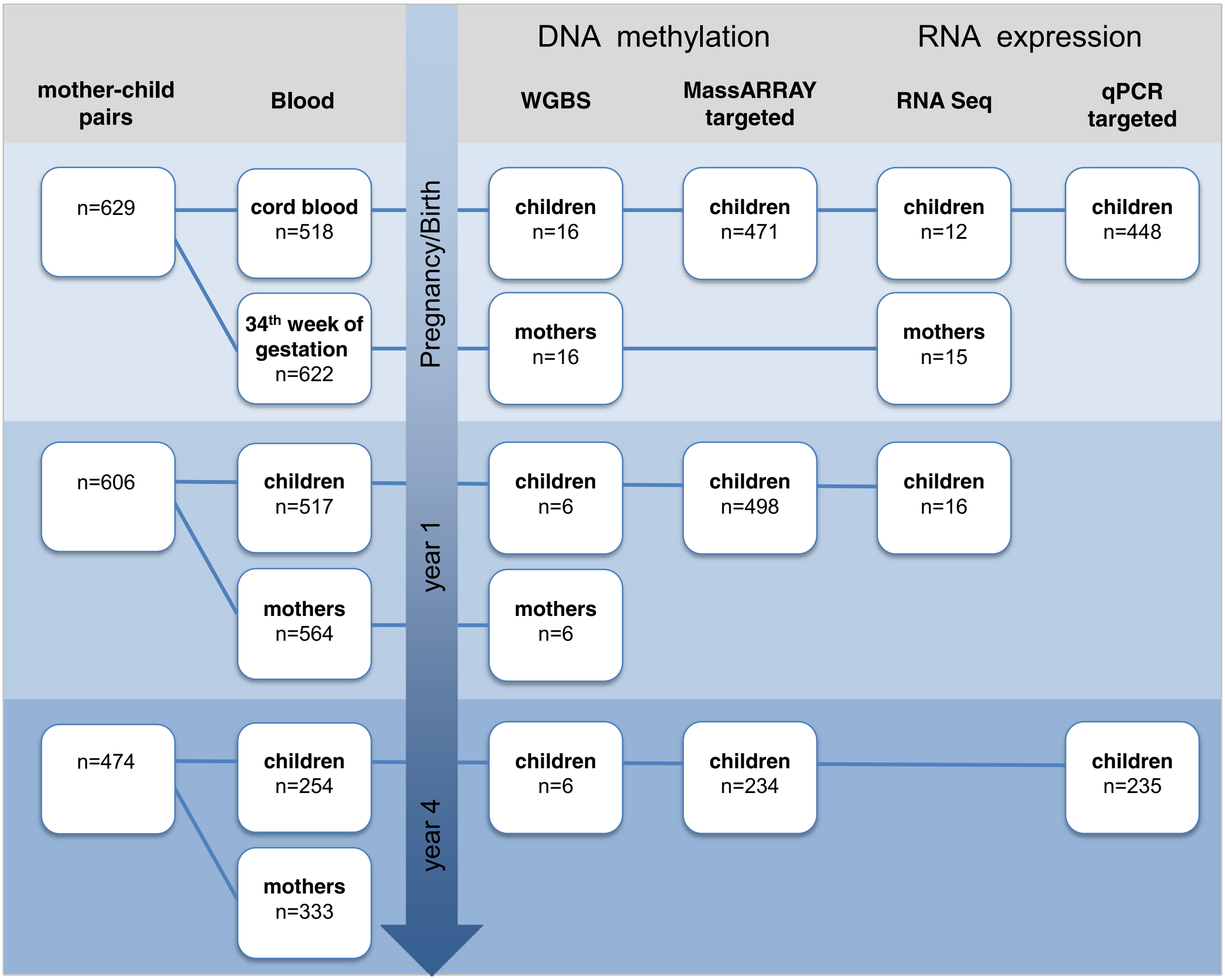

Figure EV1. Cohort and sample overview.
The diagram visualizes the number of mother–child pairs enrolled in the study together with the number of available blood samples (left). For the different experimental approaches, the number of samples analyzed is given starting at time of birth/pregnancy up to year 4 after birth (right).

© 2016 The Authors

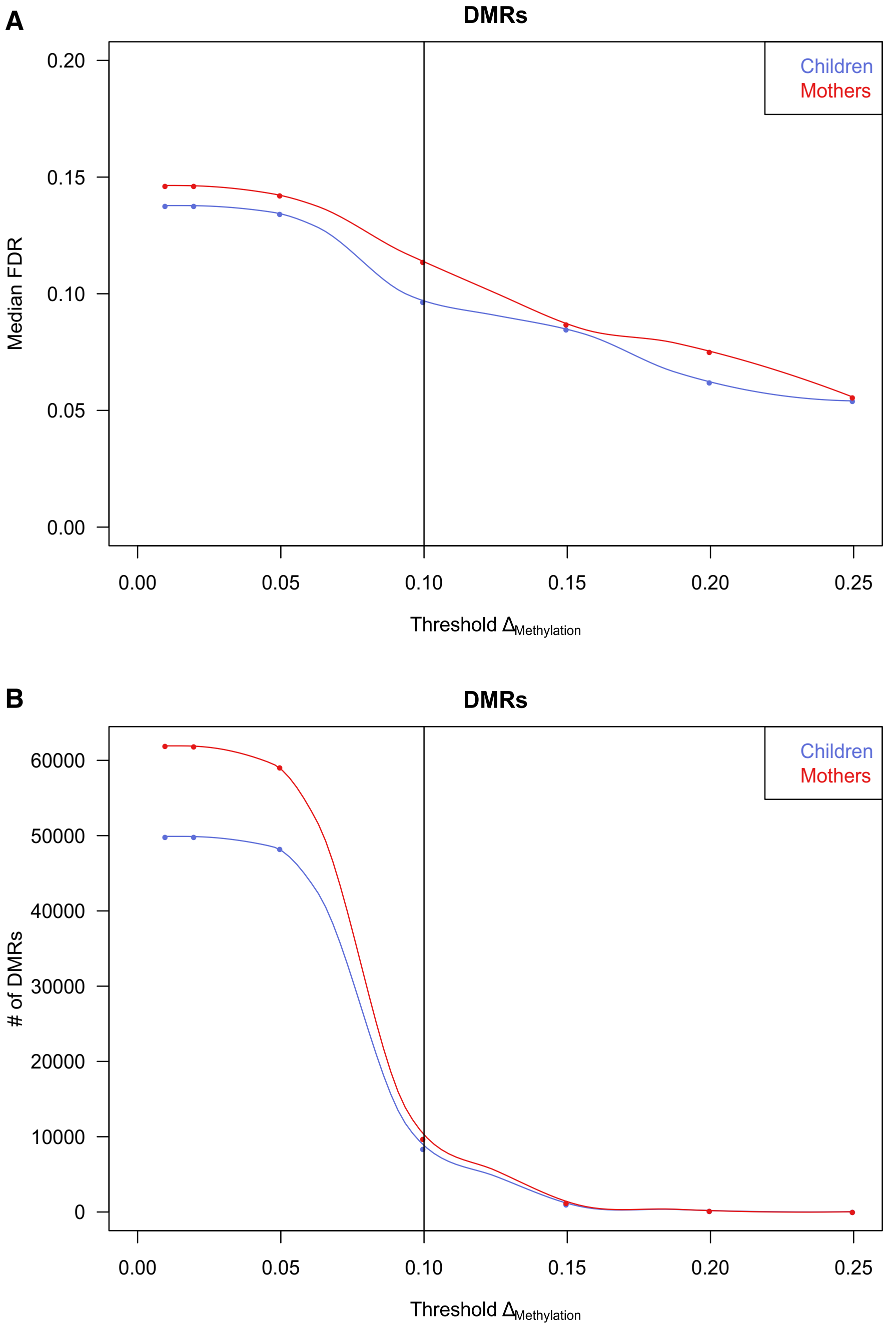

Figure EV2. FDR and number of DMRs at different methylation difference theresholds.

A Median false discovery rate (FDR) estimated by permutation analysis at different cutoffs for mean methylation changes of DMRs ($\Delta_{Methylation}$).

B Number of DMRs after filtering called at different mean methylation changes. The 10% $\Delta_{Methylation}$ cutoff is marked and indicates a fair balance between sensitivity and specificity while offering acceptable FDRs (9.7% in children, 11.4% in mothers).

Figure EV3. meQTL distinguish gDMR and ngDMR.

A–D Each DMR is associated with its most highly associated SNP within ± 5 kb, and each dot represents a DMR/SNP association. The *x*-axis shows the level of correlation between genotype and methylation level, and the *y*-axis shows the methylation difference for the DMR between the smoking and non-smoking group. DMRs associated with a CpG-destroying SNP are highlighted in green. A gDMR is defined as a DMR, which has an associated neighboring SNP with an absolute correlation above 0.6 (red vertical lines, FDR 10%), and the remaining DMRs are defined as ngDMRs (red shaded area). The effect of the 10% cutoff on the methylation difference is seen in this plot. Violin plots in (B, D) show the methylation difference in all DMRs, ngDMRs, and gDMRs.

E, F Methylation differences are shown for the various DMR stability classes, distinguishing ngDMRs (left in violin plot pairs) from gDMRs (right violin plot in each pair).

▶

 © 2016 The Authors

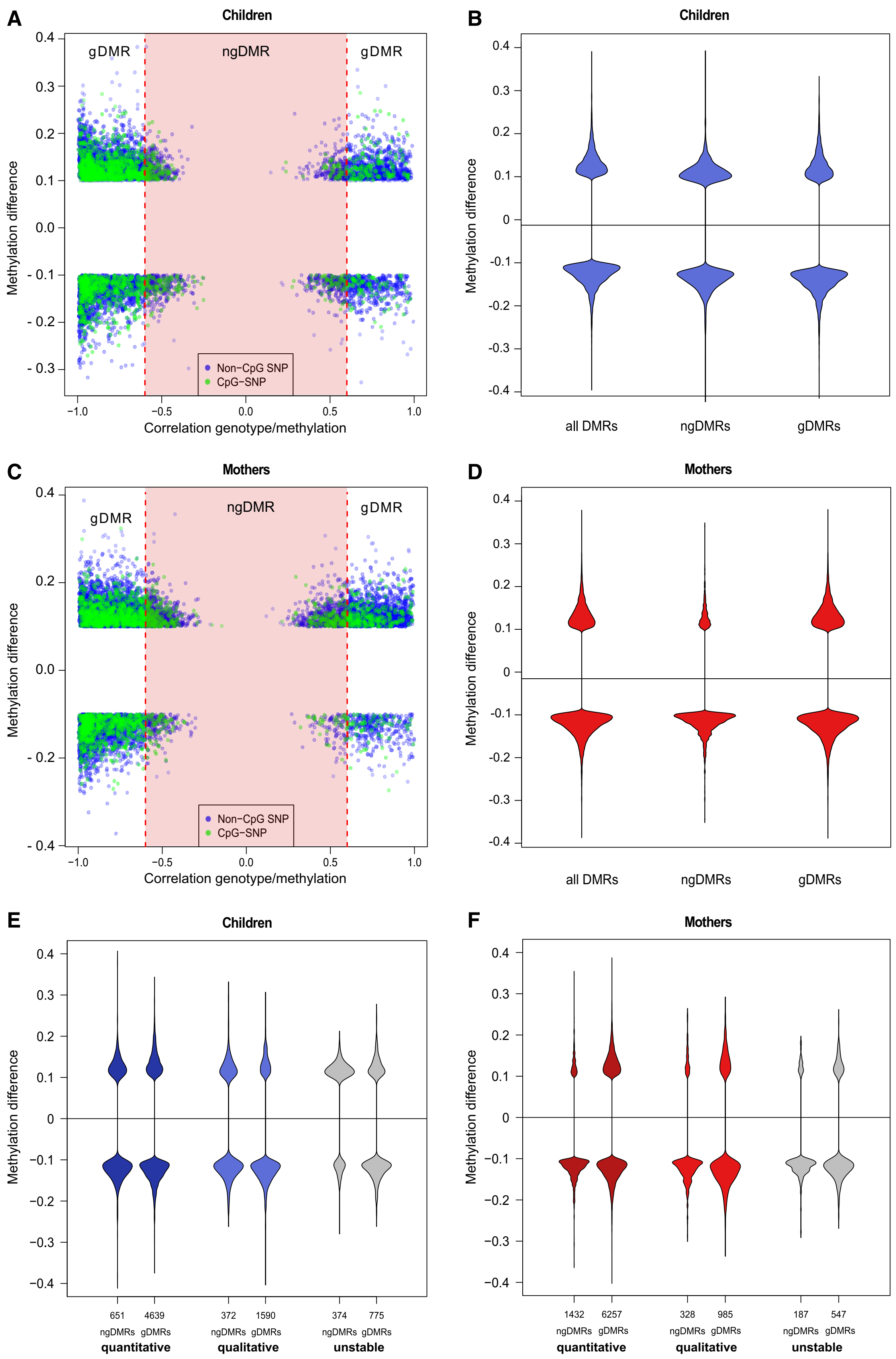

Figure EV3.

© 2016 The Authors

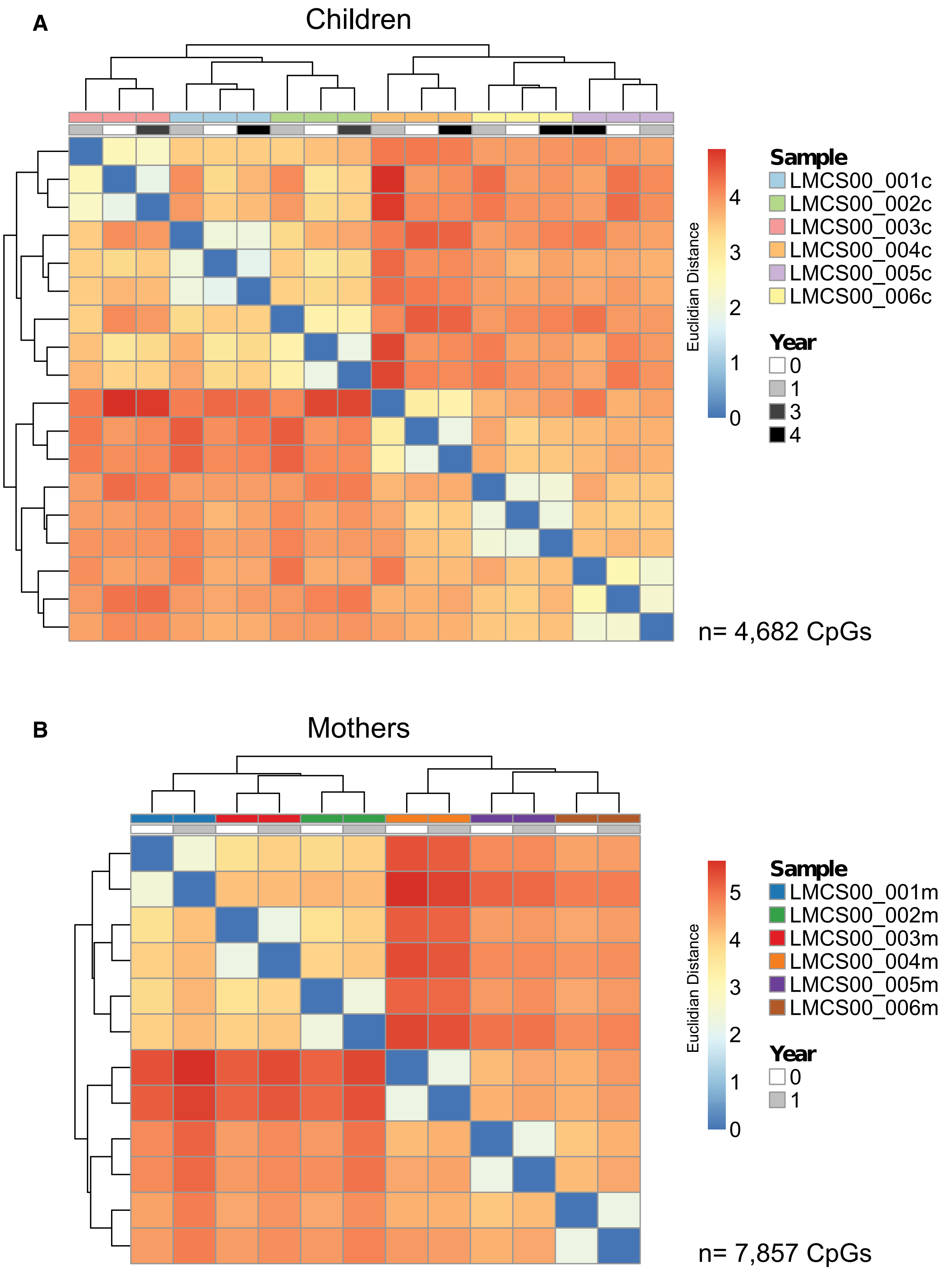

Figure EV4. Temporal DMR stability assessment by clustering DNA methylation of CpGs in ngDMRs.

A, B Hierarchical clustering (Euclidean distance) of methylation of all CpGs in ngDMRs that are not subject to SNPs in any sample. Only CpGs with a minimum coverage of 10 reads in all samples were considered. Clustering occurs by sample, not by year, suggesting high maintenance of CpG methylation levels for each sample over time. (A) Children (*n* = 4,682 CpGs), (B) mothers (*n* = 7,857 CpGs).

 © 2016 The Authors

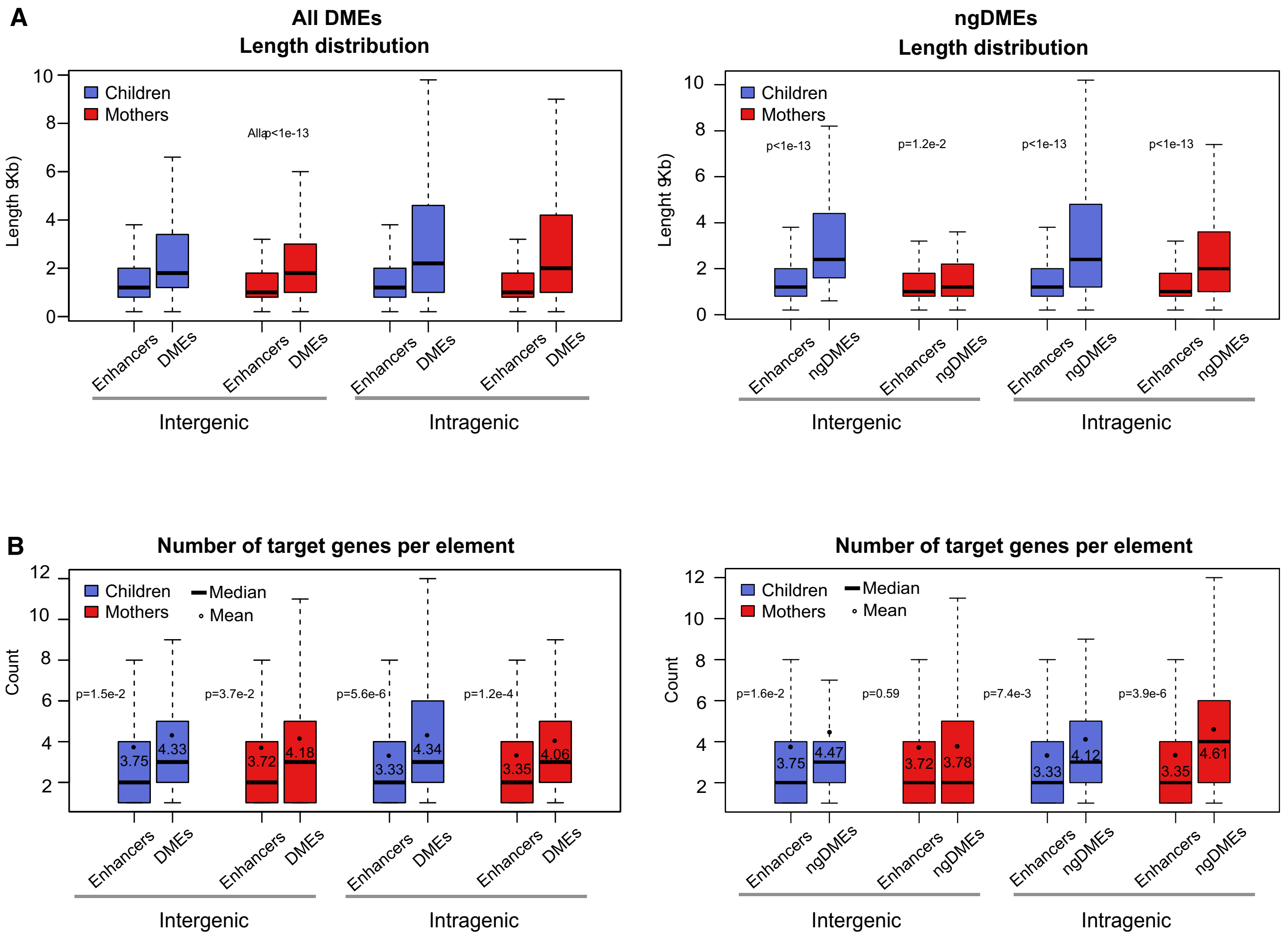

Figure EV5. Differentially methylated enhancers are regulatory hubs.

A Length distribution of enhancers (non-TSS-associated active regulatory regions) and ngDMEs in mothers and children, both inter- and intragenic, indicating a significant increase in length for differentially methylated enhancers compared to other enhancers.

B Distribution of the number of predicted target genes from the interaction datasets for the same categories as in (A). Black dots indicate the mean number. The corresponding *P*-value is indicated (Mann–Whitney test), showing a significant increase for ngDMEs, which is more pronounced in intragenic elements.

Data information: (A, B) Box plots vizualize 25/75 percentile and median, whiskers represent non-outlier range.

© 2016 The Authors

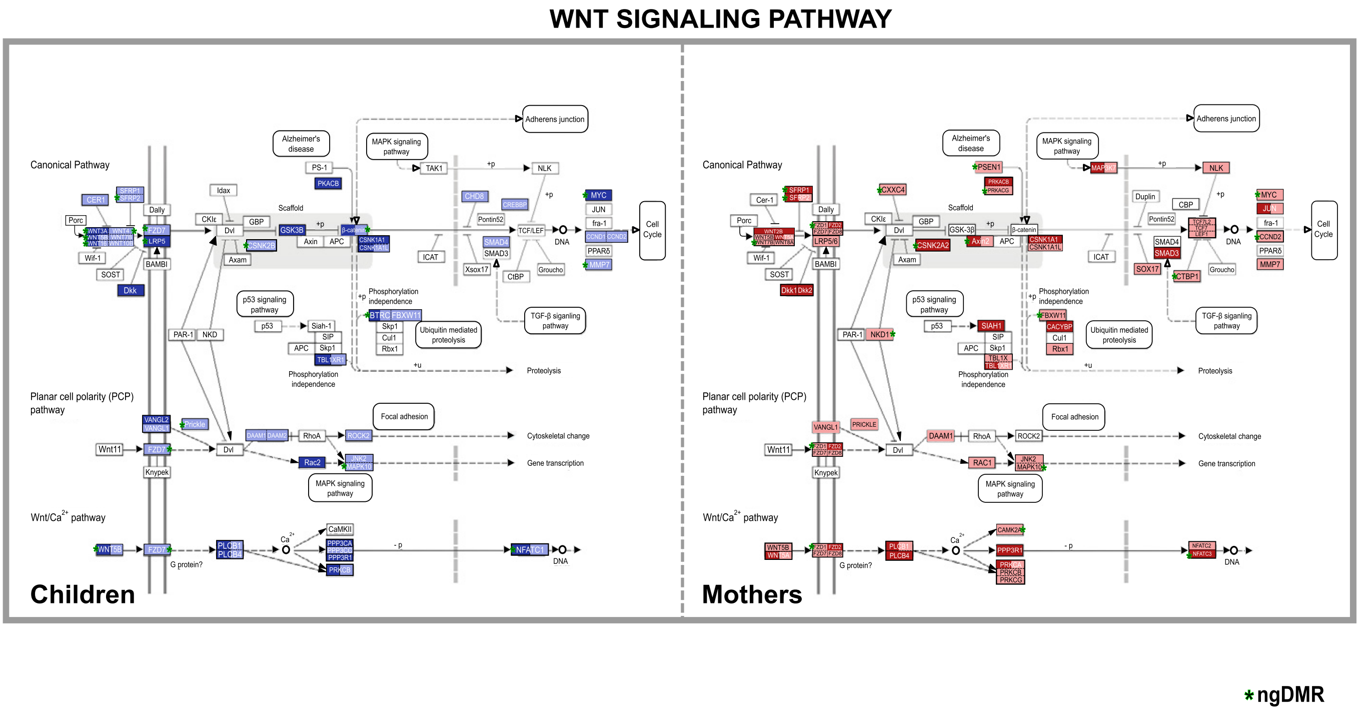

Figure EV6. Perturbation of the WNT signaling pathway.
Shown are modified outputs of the KEGG Mapper tool (v2.1, April 2014) of the WNT signaling pathway for DMRs identified in mothers and children during pregnancy and at time of birth, respectively. Each DMR assigned to a gene of this pathway is indicated in blue for children or red for mothers. The lighter hues correspond to hypomethylation, while the darker hues indicate hypermethylation. Green stars denote DMRs classified as ngDMRs.

Figure EV7. Differential methylation impacts the expression of downstream pathways.

A The bar plot shows the list of pathways that are significantly enriched among the target genes of DMRs intersecting active enhancers and promoters both in mothers and in children (enrichment *q*-value indicated on *x*-axis) and that show differential expression of genes between smoking and non-smoking samples as measured by RNA-seq in either children or mothers. Color of bars indicates significance of differential expression of genes in this pathway (see Appendix Supplementary Methods for details of the calculation). Note that the WNT pathway shows the strongest differential expression in children.

B WNT signaling genes interacting with differentially methylated enhancers (DMEs) show clustering of smokers versus non-smokers from the RNA expression profile (DME target prediction was mapped by ChiA-PET where available and nearest TSS otherwise).

▶

 © 2016 The Authors

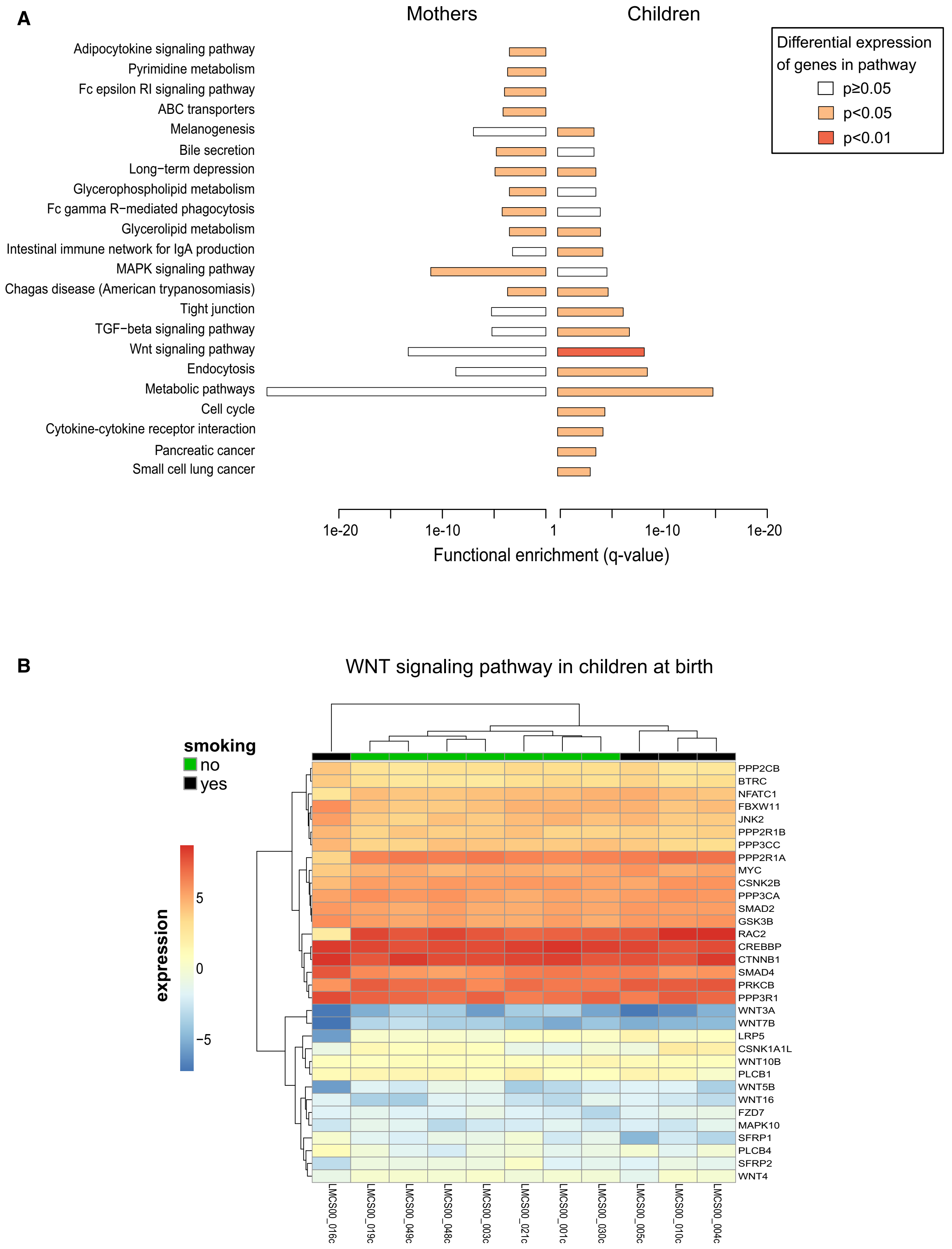
A
Mothers
Children
Differential expression of genes in pathway
p≥0.05
p<0.05
p<0.01
Adipocytokine signaling pathway
Pyrimidine metabolism
Fc epsilon RI signaling pathway
ABC transporters
Melanogenesis
Bile secretion
Long−term depression
Glycerophospholipid metabolism
Fc gamma R−mediated phagocytosis
Glycerolipid metabolism
Intestinal immune network for IgA production
MAPK signaling pathway
Chagas disease (American trypanosomiasis)
Tight junction
TGF−beta signaling pathway
Wnt signaling pathway
Endocytosis
Metabolic pathways
Cell cycle
Cytokine-cytokine receptor interaction
Pancreatic cancer
Small cell lung cancer
1e-20
1e-10
1
1
1e-10
1e-20
Functional enrichment (q-value)
B
WNT signaling pathway in children at birth
smoking
no
yes
expression
5
0
−5
PPP2CB
BTRC
NFATC1
FBXW11
JNK2
PPP2R1B
PPP3CC
PPP2R1A
MYC
CSNK2B
PPP3CA
SMAD2
GSK3B
RAC2
CREBBP
CTNNB1
SMAD4
PRKCB
PPP3R1
WNT3A
WNT7B
LRP5
CSNK1A1L
WNT10B
PLCB1
WNT5B
WNT16
FZD7
MAPK10
SFRP1
PLCB4
SFRP2
WNT4
LMCS00_016c
LMCS00_019c
LMCS00_049c
LMCS00_048c
LMCS00_003c
LMCS00_021c
LMCS00_001c
LMCS00_030c
LMCS00_005c
LMCS00_010c
LMCS00_004c

Figure EV7.

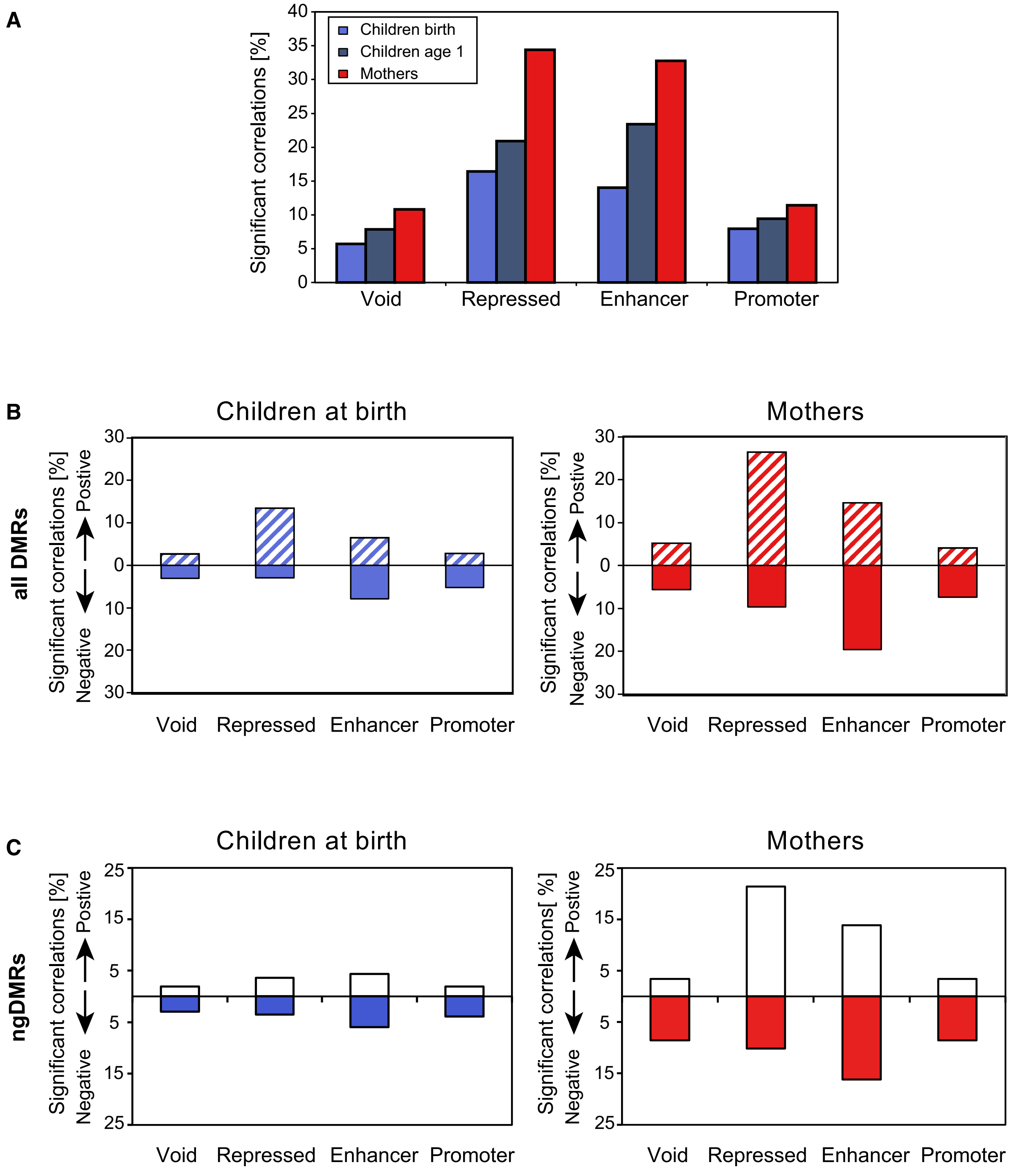

Figure EV8. Correlation of differential methylation and transcription in regulatory elements.

A Proportion of DMR-target gene pairs showing a significant correlation (Spearman's correlation test $p < 0.05$) between mean methylation and target gene expression, indicating a higher correlation level for DMRs in repressed or enhancer regions as compared to void or promoter regions. Also, correlation levels increase for children from year 0 to year 1 indicating a temporal gap between epigenetic changes and induced gene expression.

B Proportion of significantly correlated DMR-target gene pairs in the same categories as (A), split into positive/negative correlation, showing an excess of positive correlation for DMRs associated with repressed regions (containing one or both PRC-associated H3K27me3 marks as well as the repressive H3K9me3 marks), and an excess of negative correlations for active regions (enhancers and promoters).

C Proportion of significantly correlated ngDMR-target gene pairs split into positive/negative correlation in the same categories as (B).

 © 2016 The Authors

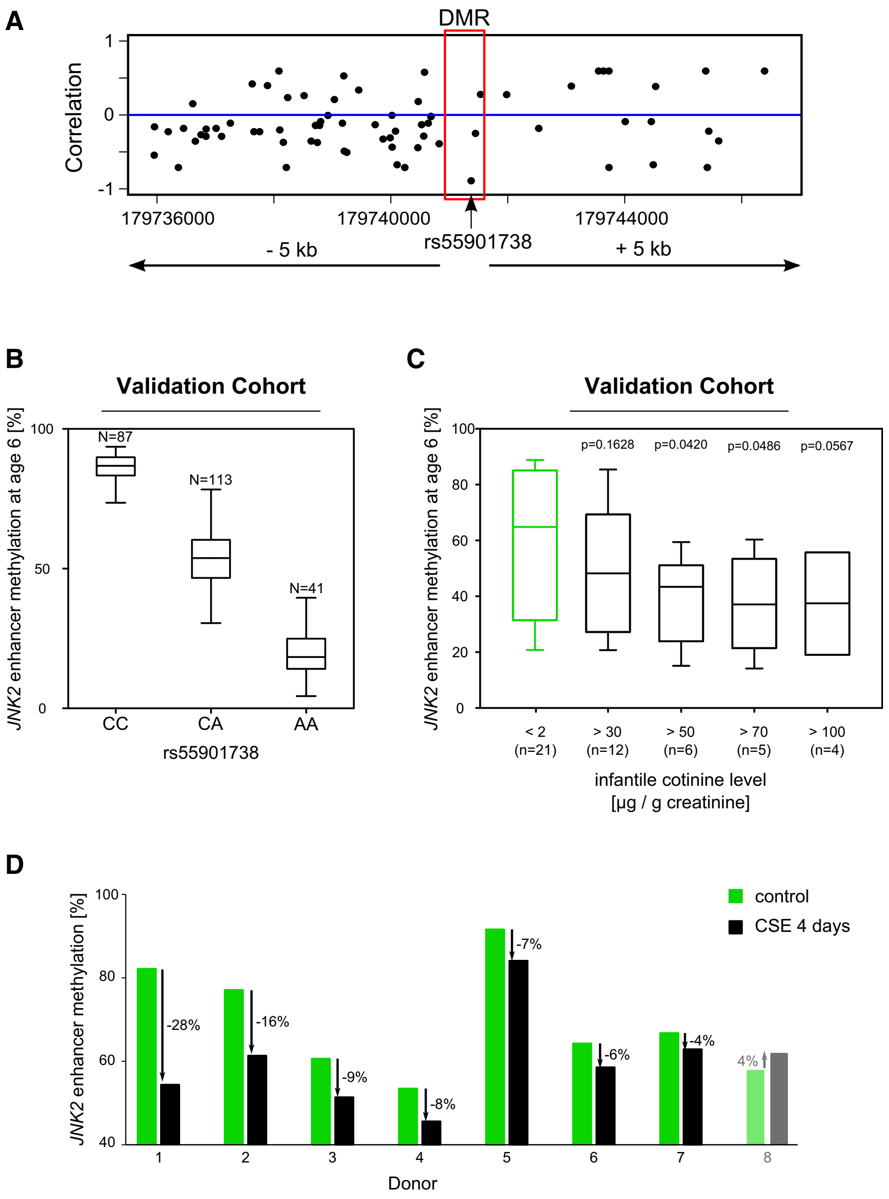

Figure EV9. Genotype and tobacco smoke exposure influence methylation of the JNK2 commuting enhancer.

A Locus around the JNK2 DMR showing for each genotyped SNP the correlation of the genotype to the methylation in the DMR region.

B Genotype at the SNP rs55901738 located inside the DME in the validation cohort (*N* = 87/113/41) indicating a clear negative correlation between the genotype and the methylation level at this locus.

C In an independent validation cohort, we confirmed in six-year-old children that cigarette smoke exposure (passive smoking) leads to a decrease of methylation in the JNK2 enhancer region. Children's urine cotinine levels inversely correlate with the JNK2 enhancer methylation level.

D Bar plots showing the loss of DNA methylation in the JNK2 enhancer region following a four-day treatment of PBMCs with cigarette smoke extract in 8 different donors.

Data information: (B, C) Box plots vizualize 25/75 percentile and median, whiskers represent non-outlier range.

© 2016 The Authors

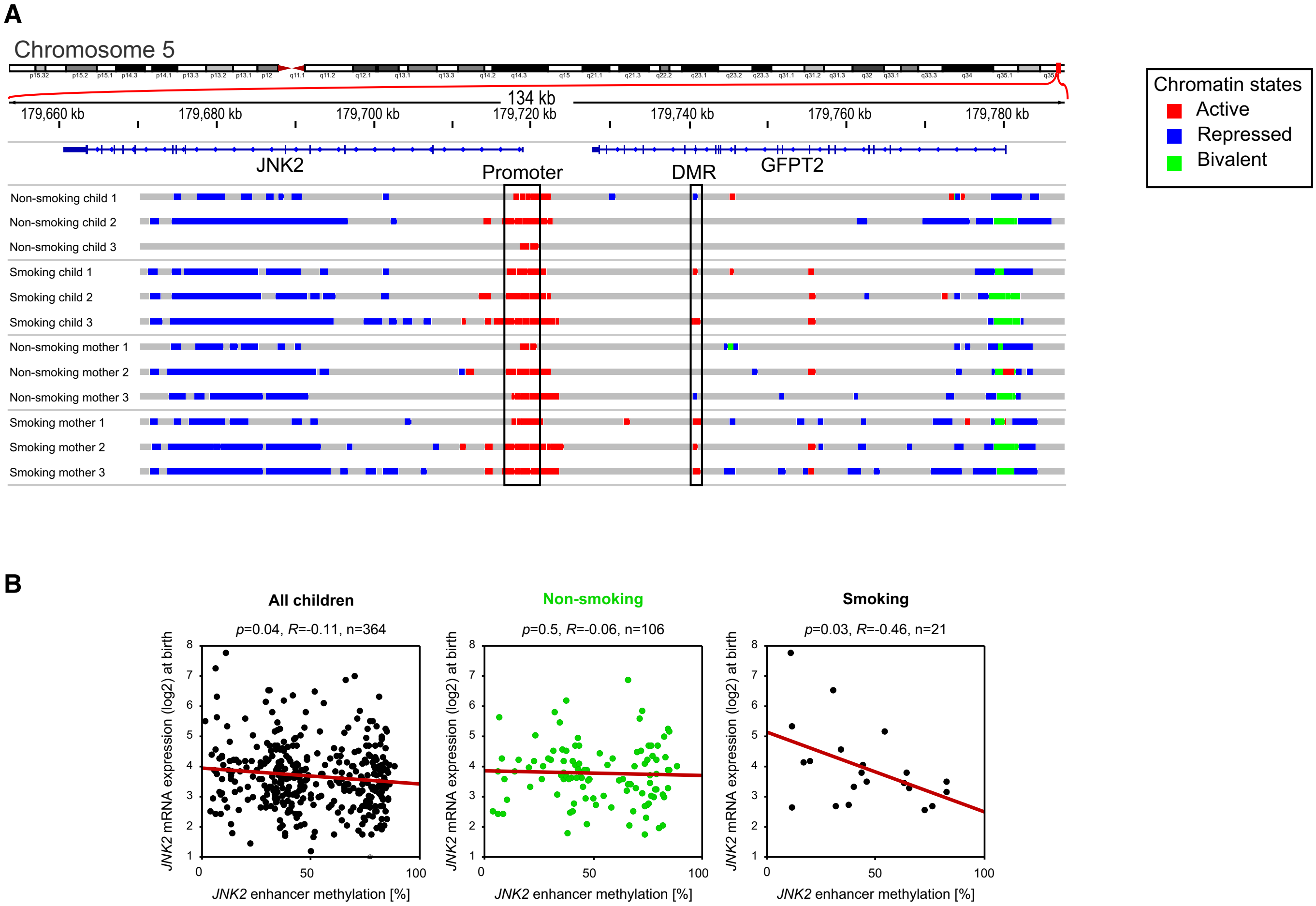

Figure EV10. Example of the commuting JNK2 enhancer located in an intron of GFPT2.

A Chromatin state comparison between non-smoking/smoking mothers and their children reveals a differentially active region in the intron of GFPT2, which corresponds to a hypomethylated gDMR in the smoking samples.

B Methylation of this region significantly, although weakly, correlates with gene expression of JNK2 (n = 364). Considering children from non-smoking (urine cotinine [µg]/creatinine [g] < 1, n = 106) or smoking mothers (urine cotinine [µg]/creatinine [g] > 100, n = 21) separately reveals that a significant correlation of methylation and JNK2 transcription is observed only for children of smoking mothers corresponding to the differentially active characteristics of this region.

 © 2016 The Authors

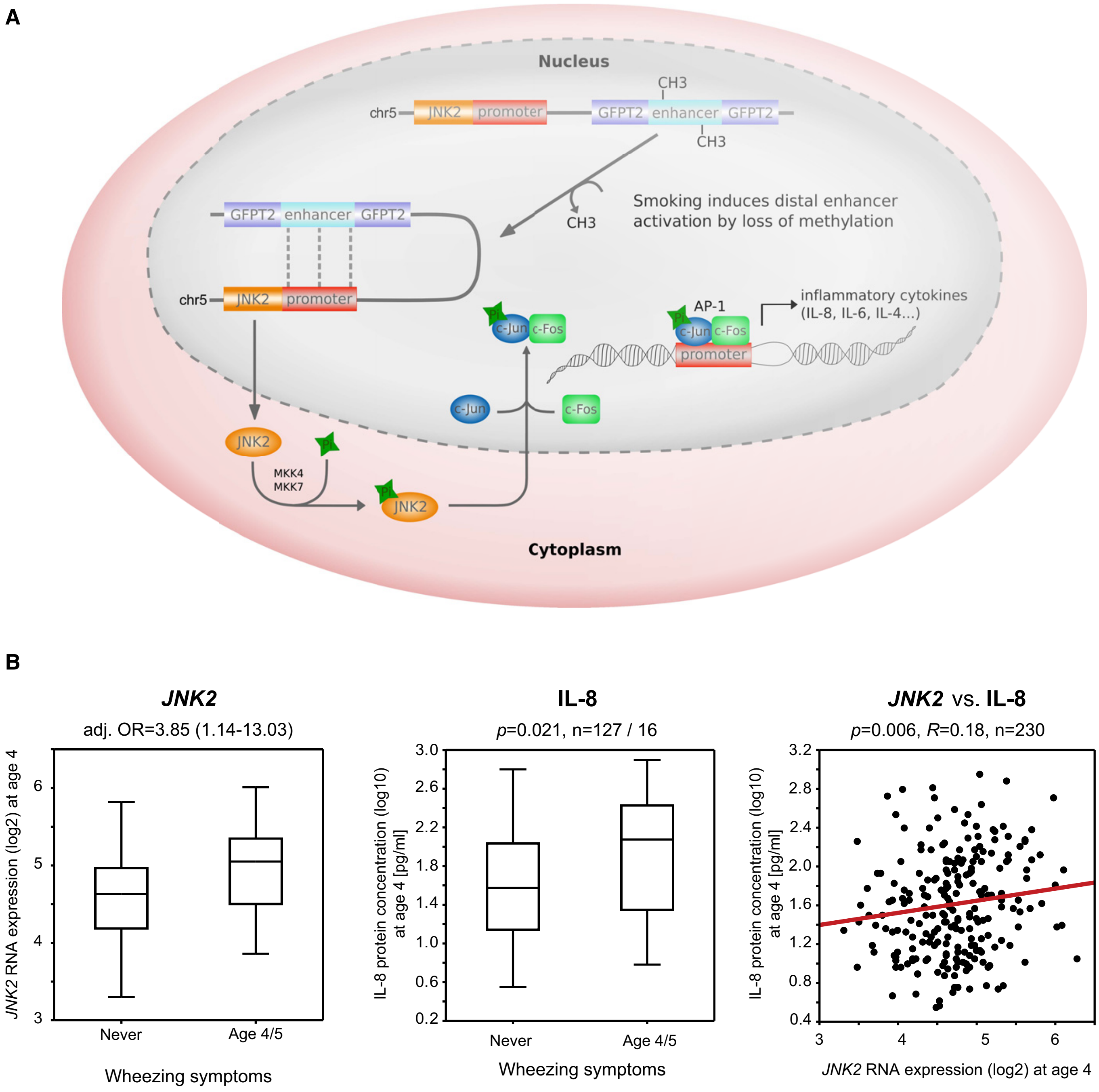

Figure EV11. JNK2 deregulation and perturbed IL-8 secretion in wheezing children.

A Loss of methylation in the differentially active JNK2 enhancer might contribute to differential JNK2 expression and altered secretion of the cytokine IL-8. JNK2 as a mediator of the early inflammatory response phosphorylates the transcription factor c-Jun that subsequently binds c-Fos, thereby forming the DNA-binding complex AP-1 (activator protein 1). AP-1 binding motifs have been identified in the promoter region of many cyto- and chemokines with IL-8 being one central downstream target.

B At age four, JNK2 gene expression and IL-8 protein concentration (measured in serum) are significantly elevated in wheezing children compared to those without any wheezing or other respiratory symptoms (Mann–Whitney test, $P = 0.037$ and 0.021, respectively). Both expression patterns correlate significantly in the entire cohort (Spearman's correlation $p = 0.006$, box plots vizualize 25/75 percentile and median, whiskers represent non-outlier range).
